# Relaxation-Enhanced Angiography Without Contrast and Triggering (REACT) for Fast Imaging of Extracranial Arteries in Acute Ischemic Stroke at 3 T

**DOI:** 10.1007/s00062-020-00963-6

**Published:** 2020-10-07

**Authors:** Lenhard Pennig, Christoph Kabbasch, Ulrike Cornelia Isabel Hoyer, Simon Lennartz, David Zopfs, Lukas Goertz, Kai Roman Laukamp, Anton Wagner, Jan-Peter Grunz, Jonas Doerner, Thorsten Persigehl, Kilian Weiss, Jan Borggrefe

**Affiliations:** 1 Institute for Diagnostic and Interventional Radiology, Faculty of Medicine and University Hospital Cologne, University of Cologne, Cologne, Germany; 2 Harvard Medical School, Department of Radiology, Massachusetts General Hospital, Boston, MA USA; 3 Else Kröner Forschungskolleg Clonal Evolution in Cancer, University Hospital Cologne, Cologne, Germany; 4 Center for Neurosurgery, Department of General Neurosurgery, Faculty of Medicine and University Hospital Cologne, University of Cologne, Cologne, Germany; 5 Department of Diagnostic and Interventional Radiology, University Hospital Würzburg, Würzburg, Germany; 6 Philips GmbH, Hamburg, Germany; 7 Department of Radiology, Neuroradiology and Nuclear Medicine, Johannes Wesling University Hospital, Ruhr University Bochum, Bochum, Germany

**Keywords:** Magnetic resonance angiography, Non-contrast-enhanced magnetic resonance angiography, Carotid arteries, ICA stenosis, Vertebral arteries

## Abstract

**Purpose:**

To evaluate a novel flow-independent 3D isotropic REACT sequence compared with CE-MRA for the imaging of extracranial arteries in acute ischemic stroke (AIS).

**Methods:**

This was a retrospective study of 35 patients who underwent a stroke protocol at 3 T including REACT (fixed scan time: 2:46 min) and CE-MRA of the extracranial arteries. Three radiologists evaluated scans regarding vessel delineation, signal, and contrast and assessed overall image noise and artifacts using 5-point scales (5: excellent delineation/no artifacts). Apparent signal- and contrast-to-noise ratios (aSNR/aCNR) were measured for the common carotid artery (CCA), internal carotid artery (ICA, C1 segment), and vertebral artery (V2 segment). Two radiologists graded the degree of proximal ICA stenosis.

**Results:**

Compared to REACT, CE-MRA showed better delineation for the CCA and ICA (C1 and C2 segments) (median 5, range 2–5 vs. 4, range 3–5; *P* < 0.05). For the ICA (C1 and C2 segments), REACT provided a higher signal (5, range 3–5; *P* < 0.05/4.5, range 3–5; *P* > 0.05 vs. 4, range 2–5) and contrast (5, range 3–5 vs. 4, range 2–5; *P* > 0.05) than CE-MRA. The remaining segments of the blood-supplying vessels showed equal medians. There was no significant difference regarding artifacts, whereas REACT provided significantly lower image noise (4, range 3–5 vs. 4 range 2–5; *P* < 0.05) with a higher aSNR (*P* < 0.05) and aCNR (*P* < 0.05) for all vessels combined. For clinically relevant (≥50%) ICA stenosis, REACT achieved a detection sensitivity of 93.75% and a specificity of 100%.

**Conclusion:**

Given its fast acquisition, comparable image quality to CE-MRA and high sensitivity and specificity for the detection of ICA stenosis, REACT was proven to be a clinically applicable method to assess extracranial arteries in AIS.

**Electronic supplementary material:**

The online version of this article (10.1007/s00062-020-00963-6) contains supplementary material, which is available to authorized users.

## Introduction

In acute ischemic stroke (AIS), imaging of the extracranial arteries is required to detect atherosclerosis and associated comorbidities, such as internal carotid artery (ICA) stenosis as well as large vessel occlusion and dissection [1].

First-pass contrast-enhanced magnetic resonance angiography (CE-MRA) using gadolinium-based contrast agents represents the standard of care for cervical arteries in stroke magnetic resonance imaging (MRI), providing high spatial resolution [2, 3]. However, CE-MRA shows limitations regarding the potential side effects of contrast agents, such as allergic reactions, nephrogenic systemic fibrosis in end-stage renal disease, and uncertain long-term effects of gadolinium deposition in the brain [4–8]. Occasionally, mistiming of image acquisition regarding first-pass contrast bolus resulting in insufficient contrast or venous contamination leads to impaired image quality in CE-MRA [9, 10]. In AIS, contrast agents might be reserved for perfusion MRI using first-pass dynamic imaging techniques [11].

Hence, several non-CE-MRA techniques have been developed in the past, with 2D/3D time-of-flight (TOF)-MRA being a possible approach for extracranial arteries; however, compared to CE-MRA, 2D/3D TOF-MRA has the disadvantages of a long acquisition time, sensitivity to respiratory and flow artifacts, inferior image quality, decreased anatomic coverage and overestimation of ICA stenosis [12–16]. Beyond TOF-MRA, different non-CE-MRA techniques have been recently proposed, with quiescent interval slice-selective (QISS)-MRA and its flow-compensated fast low-angle shot readout being one of the latest innovations to show promising results for different vascular territories, including extracranial and intracranial arteries [10, 17–20]. However, 2D acquisition and dependency on the inflow of spins from outside the saturation volume have to be considered [10, 18–20].

Recently, a novel 3D Relaxation-Enhanced Angiography without Contrast and Triggering (REACT) sequence, a combination of non-volume-selective short tau inversion recovery (STIR) and T2 preparation pulses with dual gradient echo Dixon (mDIXON XD) readout, was introduced. It combines the benefits of steady-state free precession (SSFP), such as bright-blood signal with robust fat and background suppression for flow-independent isotropic 3D non-CE-MRA [21]. While it is not suitable for intracranial MRA, REACT provides a simultaneous depiction of arterial and venous vessels and has shown encouraging results in displaying the pulmonary vasculature in congenital heart disease at 1.5 T [21, 22].

The purpose of this study was to compare the image quality of extracranial arteries and the assessment of ICA stenosis between REACT and CE-MRA at 3 T in patients with AIS.

## Material and Methods

The institutional review board approved this retrospective, single-center study (reference number: 19-1345) and waived the need for written informed consent from the patient cohort.

### Patient Population

We retrospectively reviewed our internal database for stroke MRI studies from May to July 2019. Scans were included if patients underwent a standard protocol for AIS at 3 T in clinical routine with both REACT and CE-MRA sequences depicting the extracranial arteries. Severe motion artifacts or pronounced pleural effusions led to patient exclusion.

### Imaging

All scans were performed on a clinical whole body 3 T MRI system (Philips Ingenia, Philips Healthcare, Best, The Netherlands) equipped with a standard 16-channel head and neck coil. The protocol comprised diffusion-weighted imaging in the axial and coronal planes, axial fluid-attenuated inversion recovery sequences, axial susceptibility-weighted imaging, intracranial 3D TOF-MRA, Compressed SENSE (factor 4) accelerated REACT, and Compressed SENSE (factor 6) accelerated CE-MRA. To provide a localizer for the volume placement of extracranial MRAs, a phase-contrast angiography survey served as a sagittal scout, whereas TOF-MRA yielded the axial orientation, with REACT being acquired prior to CE-MRA (Fig. 1 of the supplemental material).

For non-CE-MRA, imaging was based on a flow-independent 3D isotropic REACT sequence combining a 50 ms T2 preparation sequence and a STIR pulse with a 3D mDIXON XD (Philips Healthcare) readout [21]. The combination of T2 preparation and STIR enables the suppression of tissue with short T1 and T2, while enhancing the blood signal with long T1 and T2. For fat suppression, the mDIXON XD technique is applied. Since REACT is based on relaxation times, it can be used without any form of triggering, with data being acquired in the coronal plane. Compressed SENSE (Philips Healthcare) was used for the acceleration of image acquisition, a method providing a combination of compressed sensing and parallel imaging using SENSitivity Encoding (SENSE) [23–25]. An acceleration factor of 4 was employed, resulting in a fixed scan time of 2:46 min. Immediate image reconstruction was used. Given the known fat-water swapping artifacts of the mDIXON XD readout, water-only as well as in-phase and out-of-phase reconstructions were created [26–28].

For CE-MRA, a 3D spoiled gradient-echo T1 sequence was used. Serving as a mask, a native MRA image was acquired. Gadoteric acid (Clariscan*, *GE Healthcare, Chicago, IL, USA; 0.2 ml/kg body weight) was automatically injected into an antecubital vein at a flow rate of 2 ml/s, followed by a 30 ml saline flush. Without any triggering, acquisition in the coronal plane was started by the arrival of contrast agent in the aortic arch, as determined by a bolus tracking sequence. No table movement was conducted between bolus tracking and acquisition of the T1 sequence. No subtraction of the CE-MRA images from the native scan was performed. A Compressed SENSE factor of 6 was used, resulting in a nominal scan time of 1:08 min. Real time reconstruction was employed.

Table 1 summarizes the imaging parameters of the REACT and CE-MRA sequences. For REACT and CE-MRA, the time was noted from the beginning of acquisition until image reconstruction was completed.

**Table 1:** Imaging parameters of Relaxation-Enhanced Angiography without Contrast and Triggering (REACT) and contrast-enhanced magnetic resonance angiography (CE-MRA)

	REACT	CE-MRA
Slice orientation	Coronal	Coronal
Acquisition type	3D cartesian	3D cartesian
Acquired resolution (mm^3^)	1.5 × 1.5 × 1.5	0.63 × 0.63 × 0.63
Reconstructed resolution (mm^3^)	0.625 × 0.625 × 0.75	0.5 × 0.5 × 0.5
Field of view (mm^3^)	320 × 400 × 80	320 × 280 × 80
Flip angle	15°	40°
TR/TE1/TE2 (ms)	4.3/1.45/2.6	6.1/1.96
T2 preparation (ms)	50	–
Acceleration factor	Compressed SENSE 4	Compressed SENSE 6
Temporal resolution	–	1 s
Nominal scan time (min)	2:46	1:08

*TR* repetition time, *TE* echo time, *SENSE* Sensitivity Encoding

### Subjective Evaluation of Image Quality

Three readers with different levels of expertise in MRA (two radiologists each with 4 years of experience and one board-certified neuroradiologist with 14 years of experience) independently evaluated the MRA datasets in random order during separate sessions. Source images and maximum intensity projections (MIPs) in the coronal plane for both techniques (water-only for REACT; slice thickness of 6 mm, gap of 0 mm) were analyzed using the same IMPAX EE (Agfa HealthCare N.V., Mortsel, Belgium) workstation. Readers were aware of potential fat-water swapping artifacts in REACT and were free to choose among respective reconstructions of its source images.

The evaluation of vessel image quality was based on three distinct criteria: vessel delineation, the vessel signal, and vessel contrast to the surrounding tissue. For each criterion, a scoring scale of 1–5 was used:

1. non-diagnostic, image quality inadequate for diagnosis
2. poor, suboptimal image quality for diagnosis
3. fair, moderate image quality acceptable for diagnosis
4. good, image quality suitable for confident diagnosis
5. excellent, image quality providing highly confident diagnosis

Vessel quality was scored for the following 9 segments:

1. aortic arch/adjacent branches
2. bilateral common carotid artery (CCA)
3. bilateral ICA in the cervical (C1) segment
4. bilateral ICA in the petrous (C2) segment
5. bilateral proximal external carotid artery (ECA)
6. bilateral distal ECA (parotid space)
7. bilateral vertebral artery (V1 segment)
8. bilateral vertebral artery (V2 segment)
9. bilateral vertebral artery (V3 segment)

Additionally, investigators rated the overall presence of artifacts (blurring artifacts, banding artifacts, pulsation artifacts, and parallel imaging reconstruction artifacts) and the overall image noise with the following 5‑point scoring system: 1 non-diagnostic, 2 high impact, 3 moderate impact, 4 low impact, and 5 none.

### Objective Evaluation of Image Quality

One radiologist with 4 years of experience in MRA conducted apparent SNR (aSNR) and apparent contrast-to-noise ratio (aCNR) measurements by drawing the region of interest (ROI) in the same position on source images from REACT (water-only) and CE-MRA in the following vessels:

1. Right and left CCAs (3 cm proximal to the carotid bifurcation)
2. Right and left C1 segments of the ICA (3 cm distal to the carotid bifurcation)
3. Right and left V2 segments of the vertebral artery (4 cm distal to the transverse foramen of the sixth vertebra)

As a reference standard for background noise, an ROI was placed on the adjacent sternocleidomastoid muscle ipsilateral to the respective vessel. This intracorporeal, homogeneous tissue located close to the signal measurement was chosen as the reference standard, given the masking effect of the extracorporeal background during image reconstruction.

The aSNR and aCNR were calculated as follows:

$$\begin{aligned} \text{aSNR} &= \frac{\text{SI}_\text{vessel}}{\text{SD}\ \text{of}\ \text{SI}_\text{muscle}}\\ \text{aCNR} &= \frac{\left(\text{SI}_\text{vessel}-\text{SI}_\text{muscle}\right)}{\text{SD}\ \text{of}\ \text{SI}_\text{muscle}} \end{aligned}$$

where SI is the signal intensity, and SD is the standard deviation. For each segment, the mean value of the aSNR and aCNR for both sides was analyzed.

### Presence of Fat-water Swapping Artifacts

One radiologist with 4 years of experience in MRA assessed the acquired water maps from REACT for the presence of fat-water swapping artifacts and the corresponding signal of the in-phase image at the respective signal loss of the water map.

### Grading of Proximal ICA Stenosis

Two radiologists with different levels of expertise in MRA, 1 radiologist with 3 years and 1 board-certified neuroradiologist with 13 years of experience, independently assessed the aforementioned source images and MIPs of the extracranial MRA techniques for proximal ICA stenosis in random order during separate reading sessions. Using the same IMPAX EE (Agfa HealthCare N.V.) workstation, the following grading scale was applied:

- Grade 1: normal patency
- Grade 2: <50% stenosis
- Grade 3: 50–69% stenosis
- Grade 4: ≥70–99% stenosis
- Grade 5: occlusion

### Statistical Analysis

Statistical analysis was performed using JMP (release 14.1.0*, *SAS Institute, Cary, NC, USA), with the statistical significance set to *P* < 0.05. Quantitative measurements are indicated as the mean ± standard deviation, unless noted otherwise. The subjective image quality evaluation data are presented as medians with minimum and maximum values. To compare quantitative values and subjective scores, the Wilcoxon rank-sum test was used. The sensitivity and specificity of REACT for ICA stenosis were calculated considering CE-MRA as a reference standard since the latter provides high diagnostic accuracy [29, 30]. In cases of disagreement regarding the reference standard, a consensus diagnosis was established. A stenosis grade ≥50% was considered clinically relevant.

Interobserver agreement for the subjective evaluation of image quality was assessed using Kendall’s coefficient of concordance (Kendall’s *W*). Regarding the assessment of ICA stenosis, Cohen’s Kappa was used to evaluate the interobserver agreement of REACT and the intersequence agreement between REACT and CE-MRA considering the disease grade. The interpretation of agreement was as follows: 0.01–0.2 slight, 0.21–0.4 fair, 0.41–0.6 moderate, 0.61–0.8 substantial, and 0.81–0.99 almost perfect agreement [31].

## Results

### Study Population and Baseline Characteristics

A total of 40 patients were identified, of whom 2 had to be excluded due to severe motion artifacts in both sequences and 1 each due to artifacts in either REACT or CE-MRA, respectively. Another patient was excluded due to severe pleural effusion leading to hampered image quality in REACT. Consequently, 35 patients were included in this study (mean age 60.3 ± 21.0 years, 17 females,  range 13–86 years).

### Imaging

REACT had a fixed total acquisition time of 2:46 min. With respect to Compressed SENSE reconstruction and acquired mDIXON XD images, this amounted to an average of 3:39 ± 0:25 min of combined total acquisition and reconstruction time. CE-MRA showed a combined total acquisition (including the native scan and bolus tracking sequence) and reconstruction time of 2:59 ± 0:23 min (*P* = 0.0011).

### Subjective Evaluation of Image Quality

Three readers each evaluated 70 datasets (35 datasets each for REACT and CE-MRA), resulting in a total number of 630 analyzed arterial segments. Table 1 of the supplementary material provides the image quality scores for each imaging technique and the arterial segments regarding vessel delineation, signal, and contrast to the surrounding tissue.

**Fig. 1:**
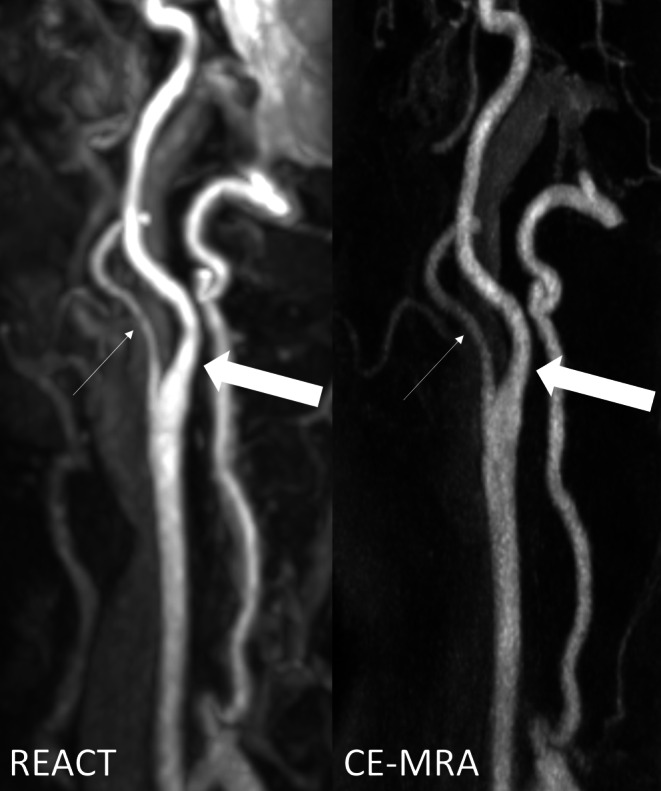
Maximum intensity projections with angulation to the left carotid bifurcation (slice thickness: 15 mm) in a 75-year-old female patient with embolic ischemia of the right cerebellum and the left precentral gyrus. Relaxation-Enhanced Angiography without Contrast and Triggering (REACT, water-only) shows higher signal and contrast as well as lower image noise, whereas contrast-enhanced magnetic resonance angiography (CE-MRA) provides better delineation of the internal carotid artery (*wide arrows* C1 segment, *thin arrows* external carotid artery)

**Fig. 2:**
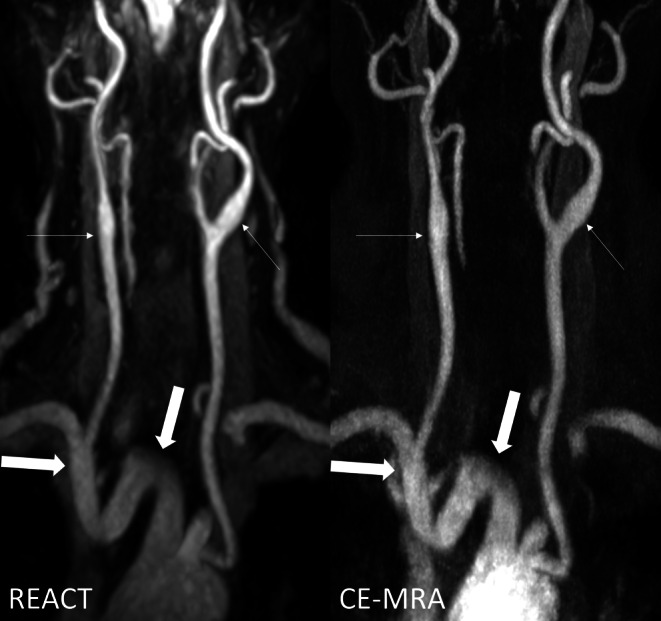
Effect of noise and pulsation artifacts on image quality in a 75-year-old female patient (same patient as in Fig. 1) with embolic ischemia of the right cerebellum and the left precentral gyrus as shown in coronal maximum intensity projections (slice thickness 15 mm). Relaxation-Enhanced Angiography without Contrast and Triggering (REACT, water-only) enables improved delineation of the branches of the aortic arch (*wide arrows*) as well as increased signal and contrast of the carotid arteries (*thin arrows*) compared to contrast-enhanced magnetic resonance angiography (CE-MRA) in which the branches of the aortic arch present pulsation artifacts and a high level of image noise leading to impaired vessel delineation

Regarding vessel signal and contrast, REACT provided superior results to CE-MRA with a higher median for signal (C1 segment: 5, range 3–5 vs. 4, 3–5; *P* = 0.004 and C2 segments: 4.5, 3–5 vs. 4, 3–5; *P* = 0.06) and contrast (C1 segment: 5, 3–5 vs. 4, 2–5; *P* = 0.346) and C2 segment: 5, 3–5 vs. 4, 2–5; *P* = 0.88) at the ICA (Figs. 1 and 2). At the aortic arch/adjacent branches, CCA, and V1–V3 segments, the same medians for signal and contrast were noted for both techniques. Of note, at the CCA, ICA (C1 and C2 segments), and V1 segment, CE-MRA demonstrated lower minimum scores than REACT for vessel contrast (Fig. 2).

**Fig. 3:**
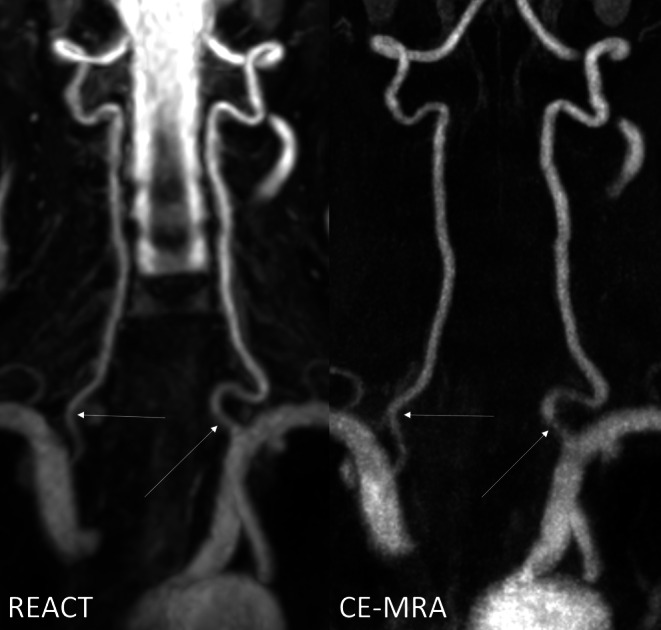
Effect of noise on image quality in an 82-year-old male patient with embolic ischemia of the right precentral gyrus as shown in coronal maximum intensity projections (slice thickness: 20 mm). In Relaxation-Enhanced Angiography without Contrast and Triggering (REACT, water-only), the V1 segments (*thin arrows*) can be sufficiently delineated, whereas image noise leads to a blurred appearance in contrast-enhanced magnetic resonance angiography (CE-MRA). The image quality of the remaining segments of the vertebral arteries is comparable between both techniques. Note the insufficiency of REACT to differentiate the V4 segments from cerebrospinal fluid

In terms of vessel delineation, CE-MRA achieved better results than REACT at the CCA (5, 2–5 vs. 4, 3–5; *P* = 0.048) and ICA at the C1 (5, 2–5 vs. 4, 3–5; *P* = 0.049) and C2 segments (5, 2–5 vs. 4, 3–5; *P* = 0.003) (Fig. 1). Considering the aortic arch/adjacent branches and the vertebral arteries, REACT and CE-MRA showed equal medians for vessel delineation. For the CCA, ICA (C1 and C2 segments), and V1–V3 segments, CE-MRA demonstrated lower minimum scores than REACT in terms of delineation (Figs. 2 and 3).

Albeit the equal medians for the ECA in the proximal and distal parts for all criteria (except for delineation of the proximal ECA: 4, 3–5 vs. 5, 2–5; *P* < 0.001), REACT provided lower values regarding the minimum score for the distal ECA for all criteria.

Regarding the overall distribution of scores for all readers combined, Table 2 provides the detailed results. REACT achieved higher values for delineation, signal, and contrast for blood-supplying vessels (BSVs; aortic arch/adjacent branches, CCA, C1 and C2 segments of the ICA, and V1–V3 segments) than for all vessels combined. CE-MRA showed that the distribution of scores was widely independent of vascular regions. For BSVs, REACT achieved good to excellent scores of 81% for delineation, 87% for signal, and 89% for contrast. CE-MRA reached good to excellent scores of 91%, 89%, and 88% of cases for these criteria, respectively. REACT provided a lower number of poor scores for these vessels than CE-MRA.

**Table 2:** Distribution of the vessel quality scores by all readers for CE-MRA and REACT in percentage with total values in brackets for all vessels (945 scores) and for blood supplying vessels (BSVs: aortic arch/adjacent branches, CCA, ICA (C1 and C2 segments), and vertebral artery (V1–V3 segments); 735 scores). Additionally, good (4) and excellent (5) ratings for REACT and CE-MRA are combined in column 9

Criterion	Modality	Vessels	1	2	3	4	5	4 + 5
Delineation	CE-MRA	All (945)	–	0.95% (9)	8.25% (78)	44.23% (418)	46.56% (440)	90.79% (858)
		BSV (735)	–	0.95% (7)	7.62% (56)	45.17% (332)	46.26% (340)	91.43% (672)
	REACT	All (945)	0.10% (1)	1.8% (17)	20.95% (198)	54.07% (511)	23.07% (218)	77.14% (729)
		BSV (735)	–	0.14% (1)	18.50% (136)	53.30% (392)	28.03% (206)	81.33% (598)
Signal	CE-MRA	All (945)	–	0.42% (4)	12.06% (114)	55.66% (526)	31.85% (301)	87.29% (827)
		BSV (735)	–	0.14% (1)	11.29% (83)	55.78% (410)	32.80% (241)	88.58% (651)
	REACT	All (945)	0.53% (5)	3.07% (29)	13.65% (129)	51.96% (491)	30.79% (291)	82.75% (782)
		BSV (735)	–	0.82% (6)	12.11% (89)	53.20% (391)	33.88% (249)	87.08% (640)
Contrast	CE-MRA	All (945)	–	1.59% (15)	12.17% (115)	46.03% (435)	40.21% (380)	86.24% (815)
		BSV (735)	–	1.77% (13)	10.07% (74)	48.03% (353)	40.14% (295)	88.17% (648)
	REACT	All (945)	0.53% (5)	2.75% (26)	13.02% (123)	58.20% (550)	25.50% (241)	83.70% (791)
		BSV (735)	–	0.41% (3)	10.75% (79)	58.01% (427)	30.75% (226)	88.76% (653)

*CE-MRA* contrast-enhanced magnetic resonance angiography, *REACT* Relaxation-Enhanced Angiography without Contrast and Triggering, *BSV* blood-supplying vessels, *CCA* common carotid artery, *ICA* internal carotid artery

There was no significant difference in the overall presence of image artifacts (REACT: 4, 3–5; CE-MRA: 4, 3–5; *P* = 0.214) (Fig. 2). REACT showed a significantly lower overall image noise than CE-MRA (4, 3–5 vs. 4, 2–5; *P* < 0.001) (Figs. 2 and 3). This difference is due to the fact that readers scored a fraction of scans from CE-MRA with high impact image noise (score of 2), whereas in the worst cases of REACT, image noise was only rated to have moderate impact (score of 3).

**Fig. 4:**
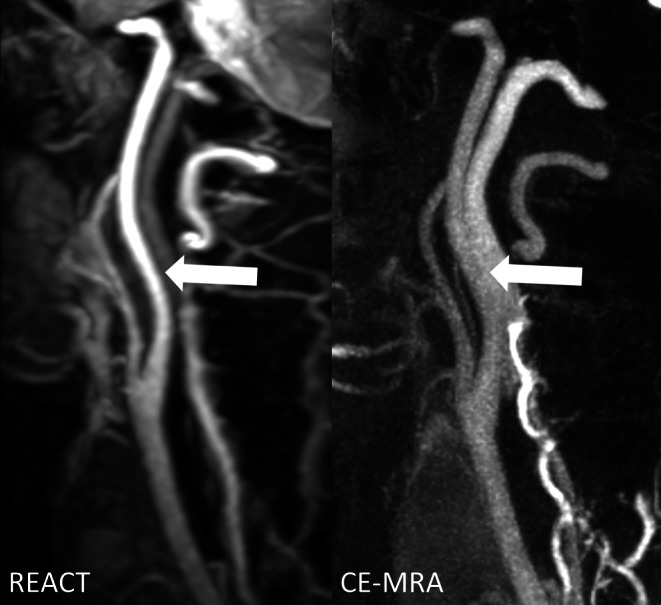
Effect of mistiming of image acquisition regarding the first-pass contrast bolus in contrast-enhanced magnetic resonance angiography (CE-MRA) in a 55-year-old male patient with pontine ischemia as shown in maximum intensity projections with angulation to the left carotid bifurcation (slice thickness: 15 mm). In Relaxation-Enhanced Angiography without Contrast and Triggering (REACT, water-only), the internal carotid artery (C1 segment, *wide arrows*) can be sharply distinguished from the adjacent internal jugular vein given its high contrast and signal, whereas in CE-MRA, venous contamination and insufficient contrast of arterial vessels lead to inferior delineation, signal, and contrast

Fig. 4 provides an additional visual comparison of the REACT and CE-MRA images, with mistimed acquisition of the latter.

### Interobserver Agreement for Subjective Image Quality in REACT and CE-MRA

The results of interobserver agreement are outlined in Table 2 of the supplementary material. There was moderate agreement (Kendall’s *W* > 0.41) for all vessel quality criteria for both methods of imaging and for noise in CE-MRA, whereas the assessment of noise in REACT and artifacts in both MRA techniques demonstrated fair agreement (Kendall’s *W* > 0.21).

### Objective Evaluation of Image Quality

Table 3 provides the detailed results regarding the aSNR and aCNR for each imaging modality and segment. REACT achieved higher aSNR and aCNR values at all analyzed segments, reaching statistical significance for the CCA and vertebral artery, whereas at the C1 segment of the ICA, no significant difference was noted (aSNR: *P* = 0.151, aCNR: *P* = 0.34). When combining all measurements, REACT provided significantly higher aSNR and aCNR values than CE-MRA.

**Table 3:** Apparent signal-to noise ratio (aSNR) and apparent contrast-to-noise ratio (aCNR) for CE-MRA and REACT. The Wilcoxon rank-sum test was used, with *P* < 0.05 indicating statistical significance

Segment	Modality	Mean ± SD	*P*-value
*aSNR*			
Common	CE-MRA	31.1 ± 8.9	<0.001
Carotid artery	REACT	51.1 ± 33.9	–
ICA	CE-MRA	59.1 ± 23.3	0.151
(C1 segment)	REACT	70.5 ± 29.6	–
Vertebral artery	CE-MRA	40.5 ± 16.1	0.011
(V2 segment)	REACT	54.1 ± 24.7	–
Combined	CE-MRA	43.7 ± 20.8	<0.001
	REACT	58.8 ± 30.8	–
*aCNR*			
Common	CE-MRA	28.1 ± 8.7	<0.001
Carotid artery	REACT	44.7 ± 29.5	–
ICA	CE-MRA	55.1 ± 22.8	0.34
(C1 segment)	REACT	63.0 ± 23.4	–
Vertebral artery	CE-MRA	37.1 ± 15.6	0.047
(V2 segment)	REACT	46.8 ± 22.3	–
Combined	CE-MRA	40.3 ± 20.2	0.002
	REACT	51.8 ± 27.8	–

*ICA* internal carotid artery, *CE-MRA* contrast-enhanced magnetic resonance angiography, *REACT* Relaxation-Enhanced Angiography without Contrast and Triggering, *SD* standard deviation

### Presence of Fat-water Swapping Artifacts

In 10 of 35 patients, fat-water swapping artifacts in REACT affecting the left subclavian artery (10 cases) and the left CCA (one case) were noted. Corresponding to the signal loss of water-only images, in-phase images provided a high vessel signal in every case (Fig. 2 of the supplementary material).

### Grading of Proximal ICA Stenosis

**Fig. 5:**
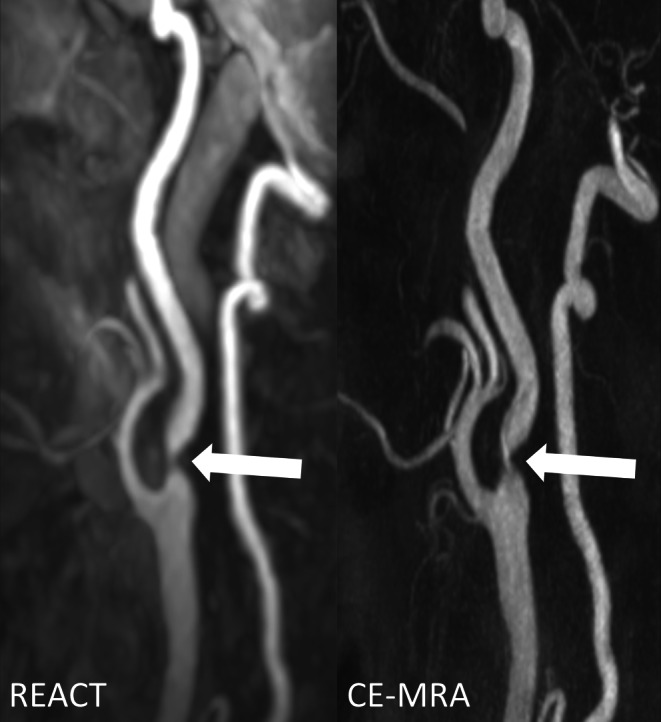
Maximum intensity projections with angulation to the right carotid bifurcation (slice thickness: 15 mm) in a 77-year-old male patient with embolic ischemia of the right precentral gyrus showing an internal carotid artery stenosis (*wide arrows*) in Relaxation-Enhanced Angiography without Contrast and Triggering (REACT, water-only) and contrast-enhanced magnetic resonance angiography (CE-MRA). In both sequences, the two readers graded the stenosis as grade 4

**Fig. 6:**
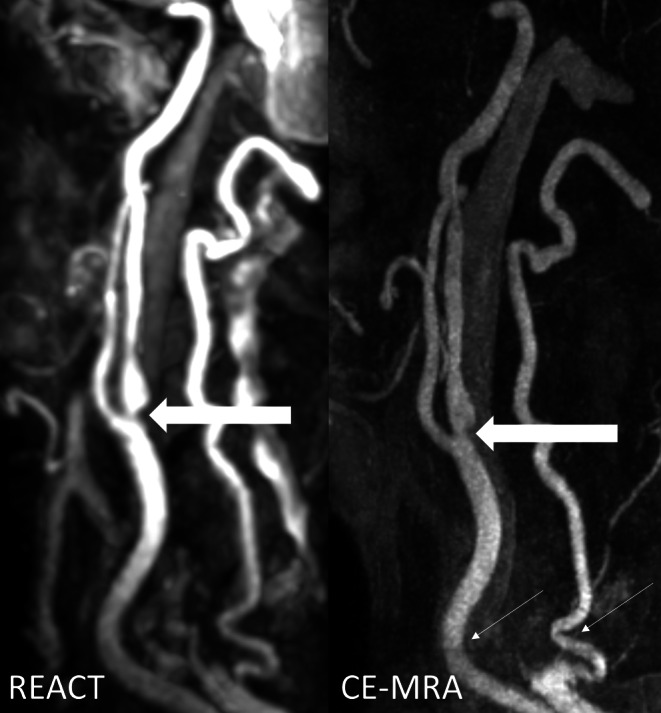
Maximum intensity projections with angulation to the left carotid bifurcation (slice thickness: 20 mm) in an 86-year-old male patient with multiple embolic ischemia of the left precentral gyrus showing an internal carotid artery stenosis (*wide arrows*) in Relaxation-Enhanced Angiography without Contrast and Triggering (REACT, water-only) and contrast-enhanced magnetic resonance angiography (CE-MRA). In both sequences, the two readers graded the stenosis as grade 3. Note the effect of image noise and pulsation artifacts on the proximal common carotid artery and V1 segment of the vertebral artery in CE-MRA, leading to impaired vessel quality (*thin arrows*)

Considering CE-MRA as a reference standard, REACT achieved a sensitivity of 90% and a specificity of 98.34% for any stenosis by both readers. Considering clinically relevant stenosis (≥50%), REACT provided a sensitivity of 93.75% with a corresponding specificity of 100%. There was almost perfect agreement for REACT between both readers (Cohen’s Kappa of 0.89). Furthermore, REACT achieved almost perfect agreement with CE-MRA regarding the disease grade (Cohen’s Kappa of 0.86) (Figs. 5 and 6).

## Discussion

In this study, we assessed a novel REACT sequence for imaging of extracranial arteries at 3 T in patients with AIS in clinical routine by comparing the image quality and grading of ICA stenosis in REACT with those in CE-MRA.

The major findings of the study are as follows: 1. In less than 3 minutes, REACT provided comparable image quality of the extracranial arteries in AIS to CE-MRA, without the use of triggering or gadolinium-based contrast agents. 2. Albeit showing inferior vessel delineation compared to CE-MRA, REACT achieved equal if not higher vessel signal and contrast for the BSVs as well as lower overall image noise outlined by subjective and objective results. 3. REACT provided high sensitivity and specificity for the detection of ICA stenosis.

In line with previous studies comparing non-CE-MRA techniques such as TOF-MRA and QISS-MRA with CE-MRA, REACT did not fully reach the exact level of image quality of high-resolution CE-MRA with inferior vessel delineation of the carotid arteries [10, 18, 19]. A possible explanation for this finding might be the slightly inferior resolution of REACT and the longer acquisition time than CE-MRA, which might impact image sharpness, e.g., due to blurring caused by physiological motion. However, the quality of the BSVs, especially regarding signal and contrast, was graded as good or excellent in the majority of cases and was pronounced at the carotid arteries. Furthermore, the subjectively described higher signal and contrast for the majority of these vessels, especially for the carotid arteries, as well as the lower overall image noise of REACT than of CE-MRA, were confirmed by objective results showing significantly higher aSNR and aCNR values for the non-CE-MRA technique. In addition to the technical differences between both MRA techniques, the higher aSNR and aCNR values with REACT may be explained by the longer acquisition time, subsequently leading to reduced noise. REACT revealed limitations in the depiction of the ECA with minor clinical relevance in the setting of AIS. Contrary to the results in carotid arteries, REACT provided equal vessel delineation of the aortic arch/adjacent branches to CE-MRA, which was occasionally hampered by pulsation artifacts. For the BSVs, REACT provided more balanced results with a lower number of segments being graded as poor quality than CE-MRA.

Considering CE-MRA as a reference standard, REACT achieved a high sensitivity (90%) and specificity (98%) for the detection of proximal ICA stenosis, especially for clinically relevant stenosis (94% and 100%, respectively). In the literature, these findings are comparable to the reported sensitivity (up to 86%) and specificity (up to 90%) of QISS-MRA for proximal ICA stenosis [10, 19]. While TOF-MRA tends to overestimate proximal ICA stenosis [14–16], almost perfect agreement was observed between REACT and CE-MRA (as reported for QISS-MRA [18, 19]) considering the disease grade, indicating the clinical potential of REACT to sufficiently detect and grade ICA stenosis without the use of gadolinium-based contrast agents.

Given the aforementioned limitations of CE-MRA, non-CE-MRA techniques are of particular interest in research and clinical practice [32, 33]. Despite its easy implementation and high spatial resolution, TOF-MRA is generally considered insufficient for imaging extracranial arteries due to its flow dependency and the long acquisition time needed to cover a large field of view as well as because its image quality is limited to the presence of horizontally directed vessel segments [12, 13, 32, 34]. Hence, there is a need for non-CE-MRA techniques yielding high image quality with a short scan time.

QISS-MRA provides a promising approach with its fast low-angle shot read-out leading to reduced sensitivity to off-resonance effects and the use of in-plane inversion allowing for adequate background suppression and high arterial-to-background contrast [10, 18, 19]. However, there are potential drawbacks due to the 2D acquisition of QISS-MRA with dependency on the inflow of spins from outside the saturation volume, which is associated with technical limitations such as anisotropic image volumes, long acquisition times, blood flow dependency, and vessel orientation relative to the imaging slices [10, 18, 19]. In contrast, the mDIXON XD read-out of REACT combines the known benefits of SSFP with the robust suppression of fat and background as well as the separation of water and fat, consequently leading to an insensitivity to inhomogeneities in the magnetic field and providing high-resolution 3D scans in a large field of view [21, 26, 35].

With the introduction of new acceleration techniques, such as compressed sensing, shorter acquisition times beyond those of the current parallel imaging techniques are feasible, especially when combining both techniques [23–25]. In this work, Compressed SENSE, which has already shown encouraging results in musculoskeletal and cardiovascular imaging, was fully integrated into the clinical system, providing image acquisition acceleration currently not achievable by compressed sensing or parallel imaging alone [22–25, 36, 37]. Given the fast acquisition time of the scan itself (less than 3 minutes) and the short reconstruction times (less than 4 minutes from the beginning of the scan to complete image reconstruction), REACT was proven to be clinically applicable in routine emergency imaging.

REACT is faster to acquire than not only QISS-MRA (scan time of up to 7 minutes) and TOF-MRA but also the CE-MRA sequence used in this study [10, 19]. Albeit having an acquisition time of approximately 1:45 minutes (when including the native scan (~5 s) and the bolus-tracking sequence (~30 s, depending on the patient’s circulation)) and a combined acquisition and reconstruction time of 3 minutes, the total time to perform CE-MRA is in fact longer than that to perform REACT when including the time needed for preparation of the patient for contrast injection. With the image quality of CE-MRA occasionally impaired due to mistiming between bolus application and data acquisition, REACT is proven to be widely user-independent and may be repeated as often as required without repetitive application of contrast agent. This is of particular relevance for patients requiring follow-up imaging [9, 10]. From an economic point of view, abandonment of contrast agents might reduce the cost of stroke MRI and facilitate clinical workflows.

Compared to 2D acquisition of QISS-MRA, REACT, similar to TOF-MRA, is able to provide an isotropic 3D readout enabling image reconstruction in all three directions of space [10, 18, 19]. The image quality of TOF-MRA and QISS-MRA may be hampered due to the dependency on blood flow and the associated sensitivity to flow artifacts [10, 12, 13, 18, 19, 32, 34]. In contrast, REACT exploits the specific relaxation properties of blood and is therefore independent of blood flow and can be acquired without any triggering, hence facilitating its use in clinical routine since head and neck imaging is generally performed without cardiac or pulse triggering [21]. Given the sufficient background suppression of the REACT sequence, an image-based navigator used to reduce swallowing motion artifacts as reported in a recent QISS-MRA study at 1.5 T is not needed [10]. Similar to TOF-MRA and QISS-MRA, REACT is acquired during free-breathing and is therefore suitable for patients unable to hold their breath [21].

The fat-water swapping artifacts of the mDIXON XD read-out with intermittent vessel signal loss in the water-only images in 10 of 35 patients may suggest a drawback of REACT [26–28]. Thus, future investigations are warranted; however, the corresponding in-phase images provided high signal for the respective vessel in each patient, clarifying the drop-out as an artifact. Whereas QISS-MRA and TOF-MRA provide scans with low or no venous contamination, the (inferior) signals of the venous vasculature and of perivascular structures may be regarded as a limitation of REACT potentially causing a crowded image [10, 18, 19, 21]. The vessel signal of REACT is T2-weighted and depends on the O_2_ saturation, leading to a higher signal and contrast of the arterial vasculature, thus enabling sufficient differentiation of arteries, veins, and adjacent soft tissues [21]. In this context, we chose a low flip angle (15°) to provide a high arterial signal. Given the selectivity of REACT for tissues with long T1 and T2, the image quality of REACT is decreased in patients with severe pleural effusions, which are not known to influence the image quality of QISS-MRA and TOF-MRA. In comparison to QISS-MRA and TOF-MRA, REACT is unable to display intracranial arteries given the high signal of cerebrospinal fluid, which also has long T1 and T2 [20, 21, 38]. This needs to be recognized as an important limitation of the sequence. Nevertheless, 3D TOF-MRA has been established as a highly reliable and standard non-CE-MRA technique for cerebral vessels with the combination of both techniques, allowing for the sufficient display of extracranial and intracranial arteries in the setting of AIS without gadolinium-based contrast agents [32, 38].

### Limitations

In addition to being a retrospective single center investigation, our study has some limitations. We did not compare REACT to digital subtraction angiography (DSA), which is considered the gold standard for imaging of extracranial and intracranial arteries [39]. The measurement of the SNR and CNR in REACT (Compressed SENSE factor 4) and CE-MRA (Compressed SENSE factor 6) may be regarded as a drawback of this study design since techniques such as parallel imaging may influence the true values of the SNR and CNR [40, 41]. However, to verify the subjective ratings of vessel signal and contrast as well as noise (and given the small difference among Compressed SENSE factors), we chose to measure the apparent values of the SNR and CNR to provide an objective evaluation of vessel signal and contrast as well as noise, with the objective results being in line with the subjective scores. Regarding the grading of ICA stenosis, a larger patient cohort may be needed to confirm the results of this study as well as to further investigate whether REACT can detect atherosclerotic plaques sufficiently and evaluate their morphology. No direct comparison to other non-CE-MRA sequences, such as QISS-MRA or TOF-MRA, was conducted in this study, which could be addressed in future investigations.

## Conclusion

This initial study indicates that REACT is suitable for routine emergency imaging, allowing fast depiction of extracranial arteries in AIS with image quality comparable to high-resolution CE-MRA as well as high detection sensitivity for ICA stenosis.

## Caption Electronic Supplementary Material

Electronic supplementary material, which includes the median subjective scores regarding vessel delineation, signal, and contrast as well as corresponding interobserver agreement of both MRA sequences. Further, scout images providing a localizer for the volume placement of extracranial MRAs and an example of a fat-water swapping artifact in REACT are presented.

## Abbreviations

AIS: Acute ischemic stroke
BSV: Blood-supplying vessels
CCA: Common carotid artery
CE-MRA: Contrast-enhanced magnetic resonance angiography
CNR: Contrast-to-noise ratio
ECA: External carotid artery
ICA: Internal carotid artery
mDIXON XD: Dual gradient echo Dixon
MIP: Maximum intensity projection
QISS: Quiescent interval slice-selective
REACT: Relaxation-Enhanced Angiography without Contrast and Triggering
ROI: Region of interest
SENSE: Sensitivity encoding
SNR: Signal-to-noise ratio
SSFP: Steady-state free precession
STIR: Short tau inversion recovery
TOF: Time-of-flight
