# Supplementary material for: Relaxation-Enhanced Angiography Without Contrast and Triggering (REACT) for Fast Imaging of Extracranial Arteries in Acute Ischemic Stroke at 3 T
Source: Clin Neuroradiol. 2020 Oct 7;31(3):815–26. doi: 10.1007/s00062-020-00963-6 (PMC8463375; doi:10.1007/s00062-020-00963-6)
Supplement: Supplementary file 1 — Electronic supplementary material, which includes the median subjective scores regarding vessel delineation, signal, and contrast as well as corresponding interobserver agreement of both MRA sequences. Further, scout images providing a localizer for the volume placement of extracranial MRAs and an example of a fat-water swapping artifact in REACT are presented. [file 62_2020_963_MOESM1_ESM.docx]

SUPPLEMENTARY MATERIAL

| Segment | Modality | **Delineation** | P-value | **Signal** | P-value | **Contrast** | P-value |
| --- | --- | --- | --- | --- | --- | --- | --- |
| Aortic arch/ | CE-MRA | **4 [2-5]** | .141 | **4 [2-5]** | <.0001 | **4 [2-5]** | .391 |
| adjacent branches | REACT | **4 [2-5]** |  | **4 [2-5]** |  | **4 [2-5]** |  |
| Common | CE-MRA | **5 [2-5]** | .048 | **4 [3-5]** | .663 | **4 [2-5]** | .582 |
| carotid artery | REACT | **4 [3-5]** |  | **4 [3-5]** |  | **4 [3-5]** |  |
| ICA | CE-MRA | **5 [2-5]** | .049 | **4 [3-5]** | .004 | **4 [2-5]** | .346 |
| (C1 segment) | REACT | **4 [3-5]** |  | **5 [3-5]** |  | **5 [3-5]** |  |
| ICA | CE-MRA | **5 [2-5]** | .003 | **4 [3-5]** | .06 | **4 [2-5]** | .88 |
| (C2 segment) | REACT | **4 [3-5]** |  | **4.5 [3-5]** |  | **5 [3-5]** |  |
| Proximal external | CE-MRA | **5 [2-5]** | <.0001 | **4 [2-5]** | .248 | **4 [2-5]** | <.0001 |
| carotid artery | REACT | **4 [3-5]** |  | **4 [2-5]** |  | **4 [2-5]** |  |
| Distal external | CE-MRA | **4 [2-5]** | <.0001 | **4 [2-5]** | <.0001 | **4 [2-5]** | <.0001 |
| carotid artery | REACT | **4 [1-5]** |  | **4 [1-5]** |  | **4 [1-5]** |  |
| Vertebral artery | CE-MRA | **4 [2-5]** | <.0001 | **4 [3-5]** | .258 | **4 [2-5]** | .008 |
| (V1 segment) | REACT | **4 [3-5]** |  | **4 [2-5]** |  | **4 [3-5]** |  |
| Vertebral artery | CE-MRA | **4 [2-5]** | <.0001 | **4 [3-5]** | .302 | **4 [2-5]** | .002 |
| (V2 segment) | REACT | **4 [3-5]** |  | **4 [3-5]** |  | **4 [2-5]** |  |
| Vertebral artery | CE-MRA | **4 [2-5]** | .003 | **4 [3-5]** | .608 | **4 [2-5]** | .398 |
| (V3 segment) | REACT | **4 [3-5]** |  | **4 [3-5]** |  | **4 [2-5]** |  |

**Supplementary tables**

Table 1: Median subjective scores regarding vessel delineation, signal, and contrast averaged as indicated by all readers for CE-MRA (contrast-enhanced magnetic resonance angiography) and REACT (Relaxation-Enhanced Angiography without Contrast and Triggering). The Wilcoxon rank-sum test was used with P<.05 indicating statistical significance. ICA=internal carotid artery.

| Criterion | CE-MRA | REACT |
| --- | --- | --- |
| Delineation | 0.43 | 0.46 |
| Signal | 0.45 | 0.44 |
| Contrast | 0.44 | 0.47 |
| Artifacts | 0.29 | 0.26 |
| Noise | 0.48 | 0.36 |

Table 2: Interobserver agreement for CE-MRA (contrast-enhanced magnetic resonance angiography) and REACT (Relaxation-Enhanced Angiography without Contrast and Triggering) for each subjective criterion of image quality assessed by Kendall’s *W*.

**Supplementary figures**

Figure 1: Sagittal tilted scout image based on phase-contrast angiography (a) and axial orientation based on prior acquired TOF-MRA (b) for the acquisition of extracranial MRAs.


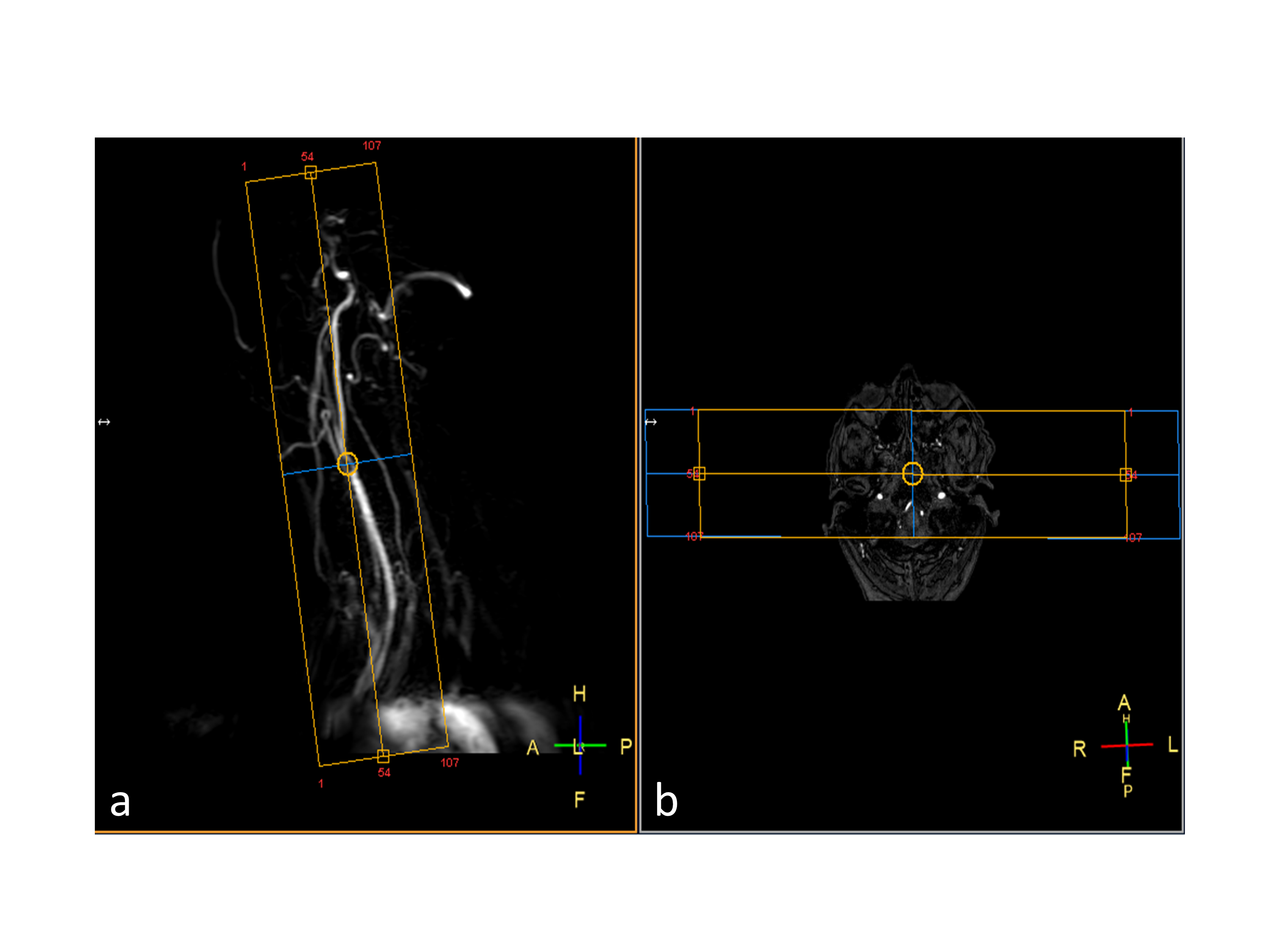

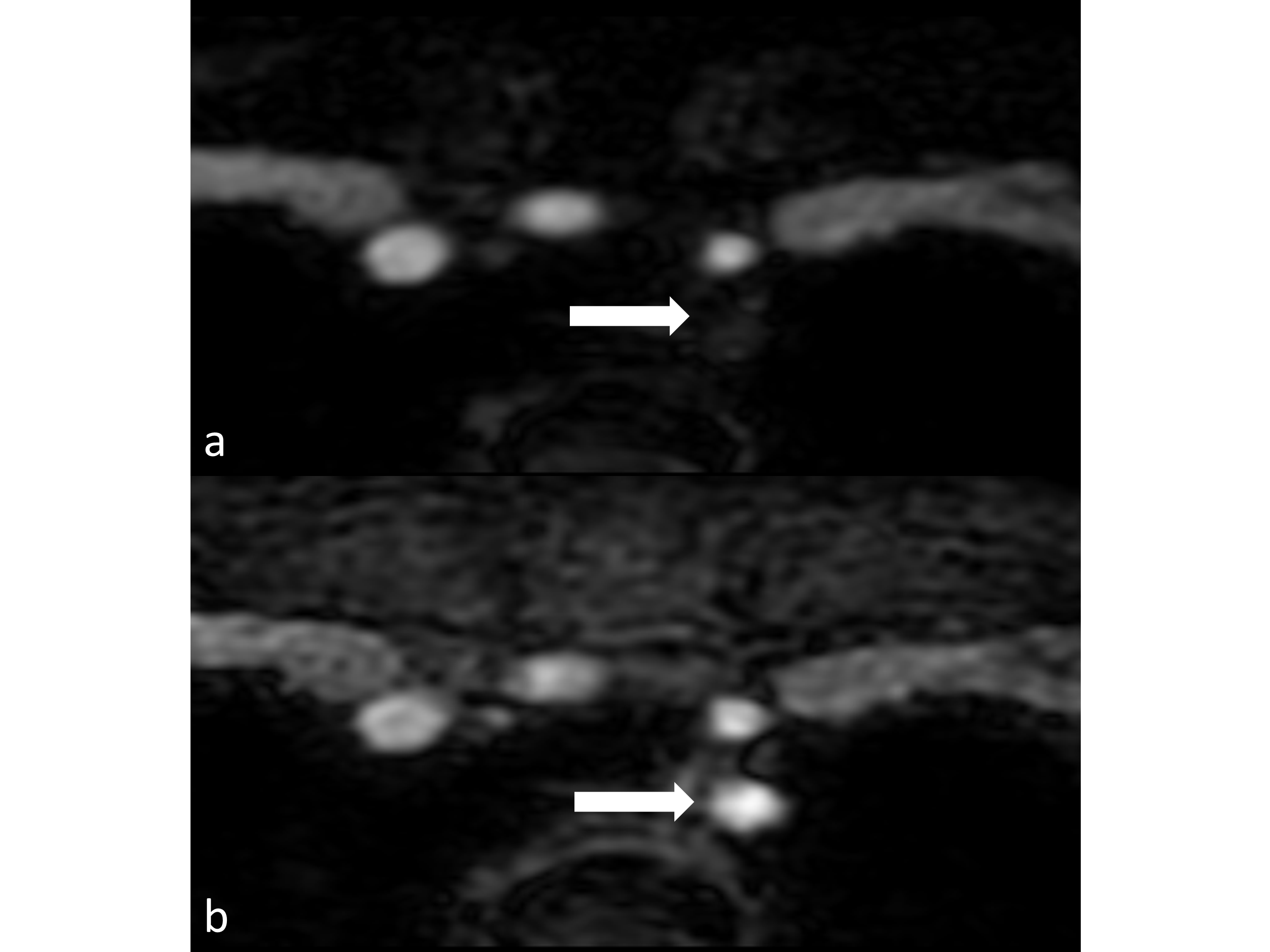


Figure 2: Example of a signal loss in water-only weighting (a) of mDIXON XD readout in axial source images from REACT with strong signal of the corresponding in-phase image (b; arrows) in the left proximal subclavian artery (1 cm distal of its origin, over a length of 2.5 cm) in a 75-year-old female patient with acute right hemispheric watershed cerebral infarction.
